# Supplementary material for: Genomic partitioning and functional dissection of inbreeding depression for stature in Brown Swiss cattle
Source: Genet Sel Evol. 2026 Jun 28;58:45. doi: 10.1186/s12711-026-01062-9 (PMC13491682; doi:10.1186/s12711-026-01062-9)
Supplement: Supplementary file 1 [file 12711_2026_1062_MOESM1_ESM.pdf]

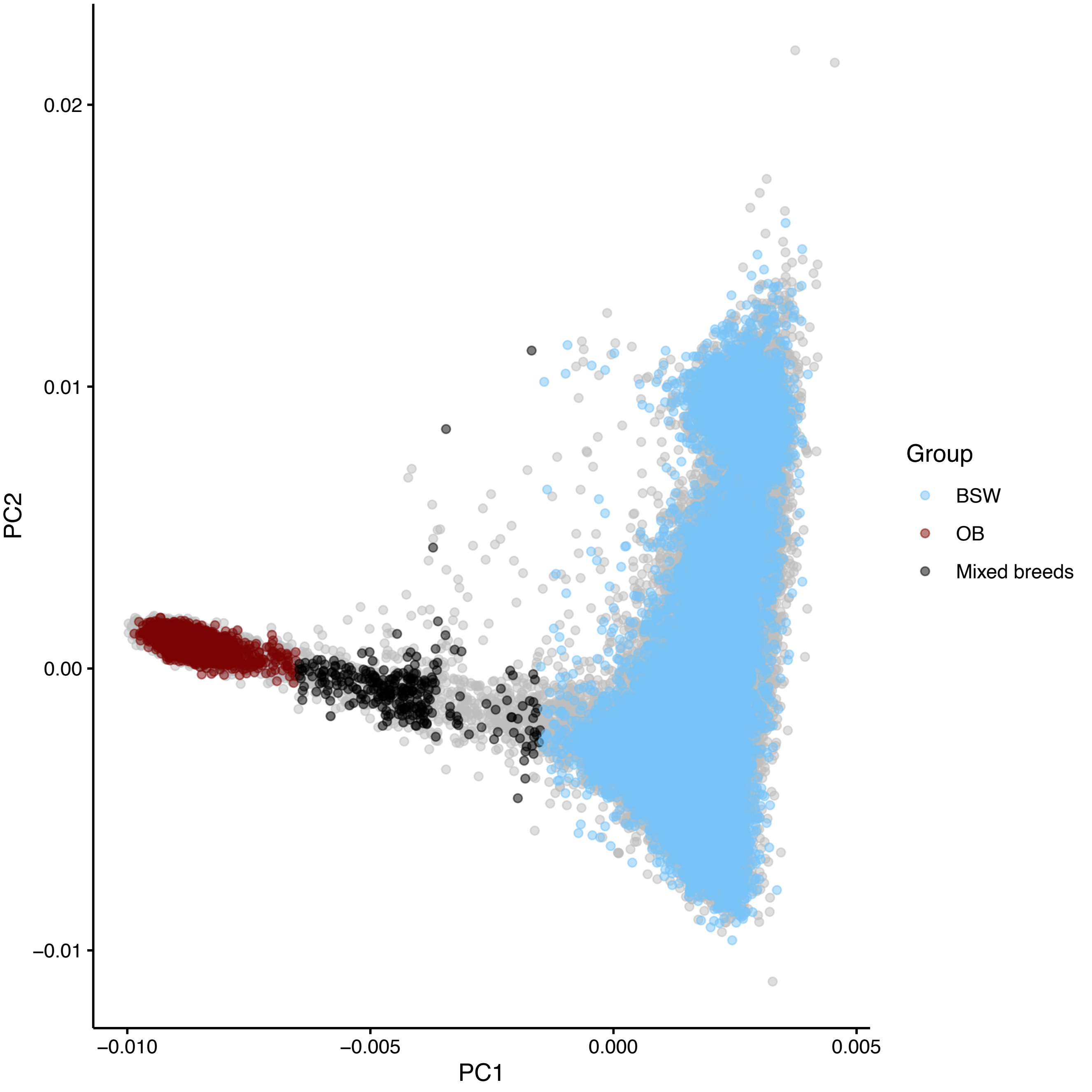

PC2
0.02
0.01
0.00
−0.01
PC1
−0.010
−0.005
0.000
0.005
Group
BSW
OB
Mixed breeds
