# Supplementary material for: Genomic partitioning and functional dissection of inbreeding depression for stature in Brown Swiss cattle
Source: Genet Sel Evol. 2026 Jun 28;58:45. doi: 10.1186/s12711-026-01062-9 (PMC13491682; doi:10.1186/s12711-026-01062-9)
Supplement: Supplementary file 2 [file 12711_2026_1062_MOESM4_ESM.pdf]

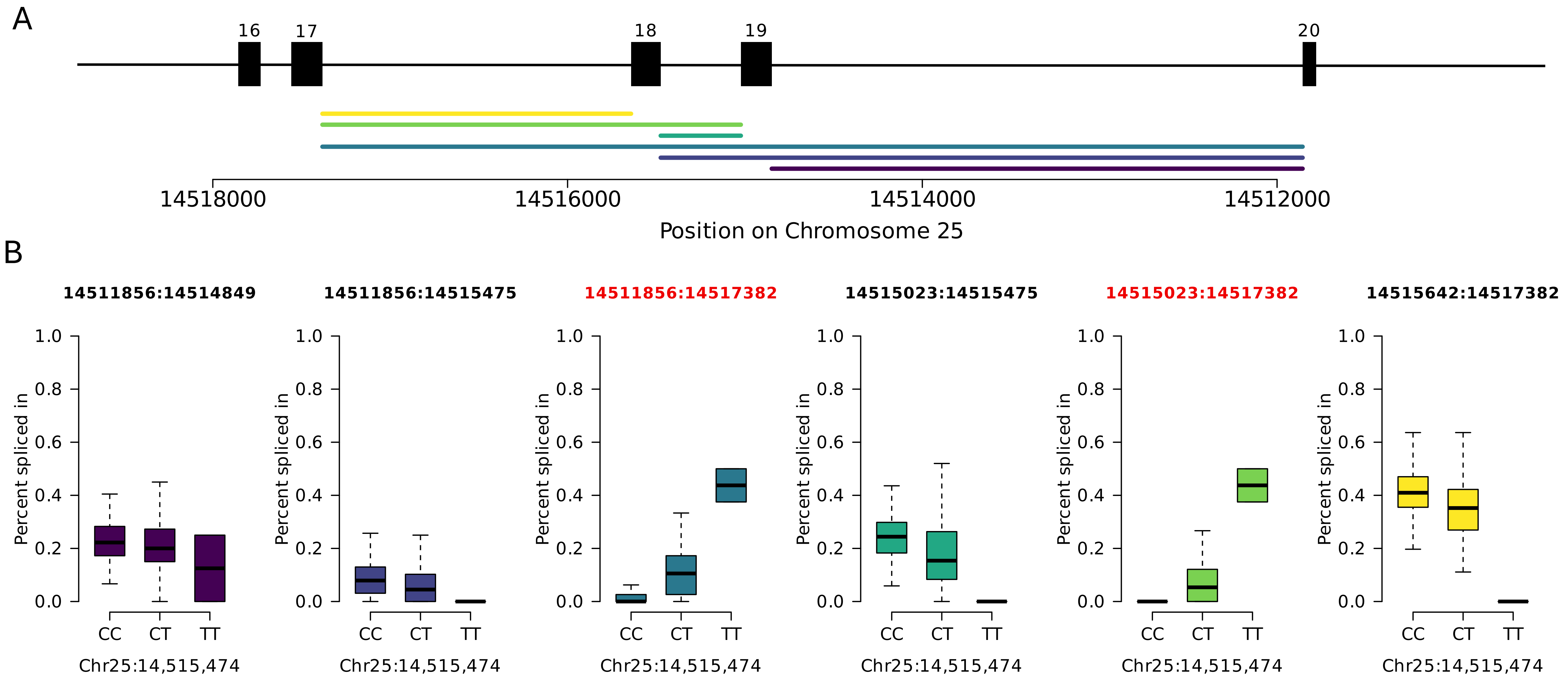

A
16
17
18
19
20
14518000
14516000
14514000
14512000
Position on Chromosome 25
B
14511856:14514849
14511856:14515475
14511856:14517382
14515023:14515475
14515023:14517382
14515642:14517382
Percent spliced in
0.0
0.2
0.4
0.6
0.8
1.0
CC
CT
TT
Chr25:14,515,474
