# Supplementary material for: Genomic partitioning and functional dissection of inbreeding depression for stature in Brown Swiss cattle
Source: Genet Sel Evol. 2026 Jun 28;58:45. doi: 10.1186/s12711-026-01062-9 (PMC13491682; doi:10.1186/s12711-026-01062-9)
Supplement: Supplementary file 3 [file 12711_2026_1062_MOESM5_ESM.pdf]

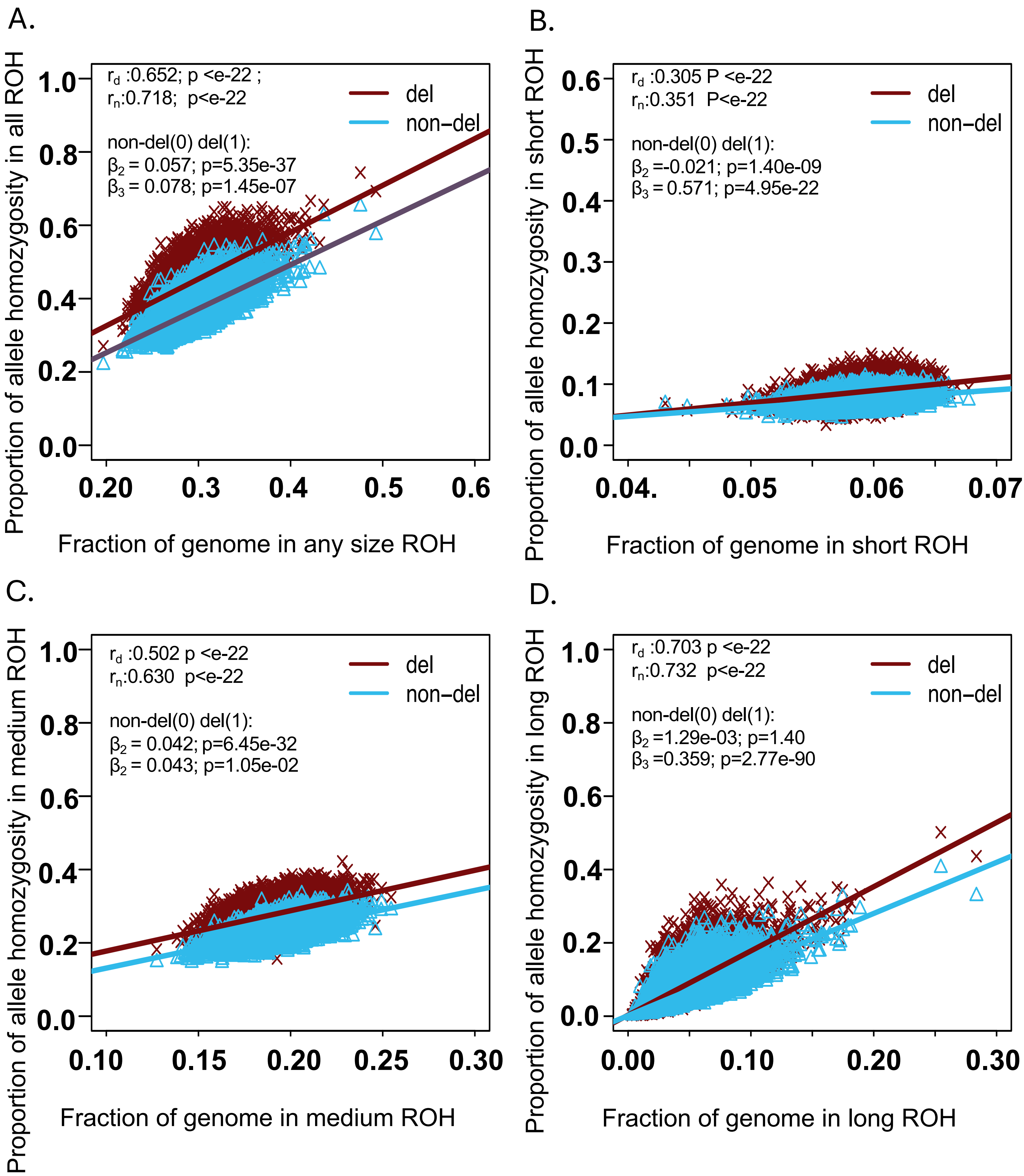

A.
r_d :0.652; p <e-22 ;
r_n:0.718; p<e-22
non-del(0) del(1):
β2 = 0.057; p=5.35e-37
β3 = 0.078; p=1.45e-07
del
non–del
Proportion of allele homozygosity in all ROH
Fraction of genome in any size ROH
B.
r_d :0.305 P <e-22
r_n:0.351 P<e-22
non-del(0) del(1):
β2 =-0.021; p=1.40e-09
β3 = 0.571; p=4.95e-22
del
non–del
Proportion of allele homozygosity in short ROH
Fraction of genome in short ROH
C.
r_d :0.502 p <e-22
r_n:0.630 p<e-22
non-del(0) del(1):
β2 = 0.042; p=6.45e-32
β2 = 0.043; p=1.05e-02
del
non–del
Proportion of allele homozygosity in medium ROH
Fraction of genome in medium ROH
D.
r_d :0.703 p <e-22
r_n:0.732 p<e-22
non-del(0) del(1):
β2 =1.29e-03; p=1.40
β3 =0.359; p=2.77e-90
del
non–del
Proportion of allele homozygosity in long ROH
Fraction of genome in long ROH
