# Supplementary material for: Genomic partitioning and functional dissection of inbreeding depression for stature in Brown Swiss cattle
Source: Genet Sel Evol. 2026 Jun 28;58:45. doi: 10.1186/s12711-026-01062-9 (PMC13491682; doi:10.1186/s12711-026-01062-9)
Supplement: Supplementary file 4 [file 12711_2026_1062_MOESM6_ESM.pdf]

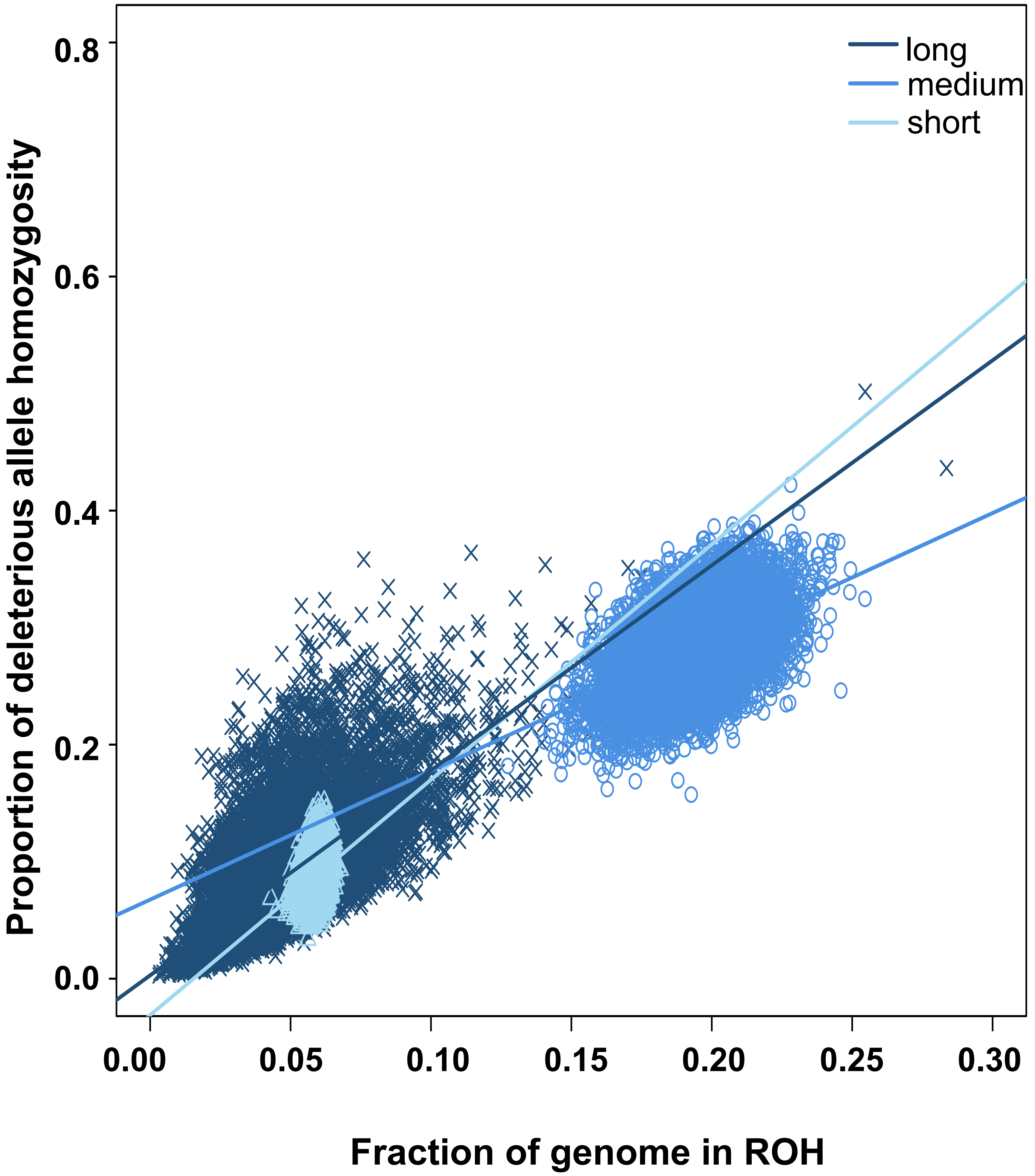

long (0) vs short (1) : β_2 = -0.033 p = 5.8e-09 ; β_3 = 0.259 p = 5.06e-03

long (0) vs medium (1) : β_2 = 0.064 p = 2.13e-82 ; β_3 = -0.651 p = 3.3e-192

medium (0) vs short (1) : β_2 = -0.098 p = 7.38e-83 ; β_3 = 0.910 p = 3.94e-29
